# Beyond behaviors: do biological intermediates improve lifestyle scoring for blood pressure?

**DOI:** 10.3389/fcvm.2025.1713086

**Published:** 2026-01-13

**Authors:** Ghadeer S. Aljuraiban, Sara Al-Musharaf, Fatima A. Almadani, Tagreed A. Mazi, Mahmoud M. Abulmeaty, Madhawi Aldhwayan

**Affiliations:** 1 Department of Community Health Sciences, College of Applied Medical Sciences, King Saud University, Riyadh, Saudi Arabia; 2 Center of Excellence in Biotechnology Research (CEBR), King Saud University, Riyadh, Saudi Arabia

**Keywords:** blood pressure, central adiposity, hypertension, lifestyle score, risk stratification, waist circumference

## Abstract

**Background:**

Hypertension prevalence is high in Saudi Arabia. Both lifestyle behaviors and biological intermediates influence blood pressure (BP); however, there is limited evidence from the Middle East on whether composite lifestyle scores that integrate both domains would capture BP variation better than behavioral metrics alone. This study aimed to examine associations of an overall lifestyle risk score (behavioral + biological) and a behavioral-only score with BP outcomes.

**Methods:**

In a cross-sectional study of Saudi adults (*n* = 1,041), we constructed (i) an overall lifestyle score (BioBeh score), including waist circumference, total cholesterol, fasting glucose, smoking, sleep, physical activity, perceived stress, and diet quality, and (ii) a behavioral-only score (Beh score), including smoking, sleep, physical activity, perceived stress, and diet. We examined the association with BP [systolic/diastolic BP (SBP/DBP)], elevated BP, and hypertension using multivariable linear and logistic regression, adjusted for various confounders.

**Results:**

Each 1-point higher BioBeh score was associated with lower SBP (−1.68 mmHg; 95% CI: −2.58, −0.79), lower DBP (−1.67 mmHg; 95% CI: −2.66, −0.68), and reduced odds of hypertension (OR 0.73; 95% CI: 0.63, 0.85), but not with elevated BP. No associations were found with the Beh score. Waist circumference was the strongest individual component [SBP −3.80 (−4.70, −2.91); DBP −3.31 (−4.30, −2.32) mmHg; hypertension OR 0.57; 95% CI: 0.49, 0.66].

**Conclusions:**

In Saudi adults, a biologically enriched lifestyle score captures BP variation and hypertension risk more robustly than behavior-only metrics. These findings support risk stratification and prevention strategies that emphasize central adiposity alongside basic lipid and glucose profiles.

## 1 Introduction

Hypertension remains one of the leading preventable causes of premature morbidity and mortality worldwide (1). The number of people living with hypertension worldwide [blood pressure (BP) of ≥140 mmHg systolic or ≥90 mmHg diastolic or on medication (2)] increased between 1990 and 2019, rising from 650 million to 1.3 billion individuals (3). A substantial burden also exists in Saudi Arabia, mirroring this global trend (4, 5). A systematic review and meta-analysis of 29 studies reported hypertension prevalence ranging from 15% to 33% in national community samples from Saudi Arabia (4). National household survey data suggest that 9% of individuals aged 15 years and older had hypertension, with prevalence ranging from 6% to 10% across Saudi regions and increasing drastically with age (6). Likewise, the World Health Organization (WHO) 2023 hypertension country profile for Saudi Arabia reported gaps in diagnosis and management (5), which were echoed in the systematic review and meta-analysis showing generally low rates of awareness, treatment, and control (4). These findings highlight the need for improved national hypertension prevention and screening strategies (5). The WHO Global Report on Hypertension reiterated the consequences of missed opportunities for prevention, detection, and treatment and called for population-level strategies that target modifiable risk factors (7). As such, comprehensive lifestyle modification is now emphasized as a cornerstone of BP prevention and management across the life course in current guidelines (8).

Evidence has shown that lifestyle behaviors related to diet quality, physical activity, tobacco smoking, sleep, and psychosocial stress are associated with BP levels and hypertension risk (9–15). This is particularly evident in Saudi Arabia, which is undergoing rapid urbanization accompanied by lifestyle and dietary transitions (16), including adapting Western-style dietary habits, with an increased availability and intake of energy-dense processed foods and sugar-sweetened beverages (17). This is combined with a general trend toward physical inactivity, with recent national and policy analyses indicating that only about 30% of Saudi adults meet the recommended physical activity levels (18).

Researchers have combined behavioral and biological factors to develop composite lifestyle scores rather than relying on single exposures to reflect the joint effects across correlated behaviors and biological factors. These lifestyle scores provide a single summary metric of cardiovascular health and improve risk stratification relevant for prevention and clinical screening (19). Frameworks such as the American Heart Association (AHA) Life's Essential 8 score have formalized the joint contributions of behaviors (diet, physical activity, nicotine exposure, sleep) and biological factors (adiposity, lipids, glucose, BP) to cardiovascular health, with higher scores associated with lower incident hypertension and better cardiovascular outcomes (19, 20). Building on that concept, in prior work, we developed a BP-specific composite that began with a “basic” score [waist circumference (WC), smoking, total cholesterol] and then added additional lifestyle components sequentially to test whether broader coverage improved prediction performance (21). We applied this tool to the UK Airwave cohort of >40,000 adults and demonstrated that higher lifestyle scores were associated with lower systolic and diastolic BP and lower odds of hypertension (21). Although the tool showed promise in the UK cohort, its applicability in other regions has not been examined (21). Region-specific investigation is warranted in Saudi Arabia, given rapid urbanization, dietary transition, physical inactivity, and high central adiposity, in addition to persistent gaps in awareness, treatment, and control. Findings from such investigations can also guide the development of low-cost screening tools (behavior only) as compared to enhanced screeners (combining anthropometry and biological indices) to inform targeted prevention strategies in the Saudi context.

Similar a priori lifestyle scores that combine behavioral and biological components are evolving in other Middle Eastern settings for related outcomes (e.g., obesity phenotypes). These studies demonstrate that the components can be operationalized using routinely collected data in the region and that the resulting scores yield gradients across clinically meaningful phenotypes, feasible to implement and straightforward to interpret (22). Despite the high cardiometabolic burden in the region (23), no research has yet applied our BP lifestyle risk score or any comparable score in Saudi Arabia. Most local studies have focused on prevalence related to a single risk factor, rather than identifying a combined lifestyle/biological phenotype (4–6). The objective of the present study is to investigate the association of a behavioral-only lifestyle risk score [(Beh score) including smoking, physical activity, sleep, diet, and stress] and a combined score of behavioral and biological indices [(BioBeh score) including WC, fasting blood glucose, and total cholesterol] with systolic and diastolic BP and the odds of hypertension among Saudi adults. This design allows quantification of the incremental value of biological intermediates beyond behaviors, clarifying the relative and joint contributions of lifestyle behaviors and their biological correlates to BP profiles in a Middle Eastern population. Risk-stratified results can be operationalized locally by prioritizing individuals with unfavorable metabolic phenotypes for intensive lifestyle counseling, closer BP monitoring (including ambulatory BP), and timely pharmacologic evaluation, while those with favorable profiles receive routine follow-up.

## 2 Methods

### 2.1 Study design

This cross-sectional study was conducted in Riyadh and Almadinah, Saudi Arabia to examine the lifestyle factors associated with BP in Saudi adults. The study was approved by the KSU Institutional Review Board (KSU-IRB-21-314). All participants provided written informed consent before enrollment.

The present work used data to construct two lifestyle scores: lifestyle score-BioBeh and lifestyle score-Beh, following previously published lifestyle-score concepts, including the one developed by our team (20, 21). The scores were then examined in terms of their relationship to BP and prevalent hypertension.

### 2.2 Participants

Recruitment invitations were distributed via social media platforms, local primary care clinics, and King Saud University (KSU) email lists to invite staff, students, and their family members to participate. Eligible participants were Saudi nationals aged ≥18 years. Exclusion criteria included pregnancy, lactation, malignancy, chronic renal or hepatic failure, malabsorption syndromes, incomplete data, and current use of glucocorticoids or weight loss medications. Interviewers were trained to review each questionnaire item in real time and prompt participants to complete missing answers. Participants with missing data on blood pressure, any lifestyle score component, or key covariates were excluded. All analyses used complete cases only (Supplementary Figure S1).

Participants completed two encounters. First, screening and on-site assessment occurred at KSU portable clinics set up in shopping malls and at the study labs in the cities of Riyadh and Almadinah. After consent was obtained, trained interviewers collected standardized anthropometric information. In addition, interviewer-administered questionnaires captured sociodemographic data, health history, diet, physical activity, sleep, and perceived stress. Second, participants attended scheduled fasting laboratory visits at private medical laboratories for blood collection.

### 2.3 Sample size calculation

Using Saudi estimates of hypertension prevalence of approximately 14% and targeting a detectable odds ratio (OR) of 1.30 (6) per 1-SD higher lifestyle risk score, with 80% power (*β* = 0.20) and two-sided *α* = 0.05, the standard formula for a continuous predictor in logistic regression indicated *n* = 900 participants. Allowing for 10% dropout for incomplete data yielded a target of *n* = 1,000.

### 2.4 Lifestyle score development

Data were collected using structured interviewer-administered questionnaires, dietary recalls, clinical blood sampling, BP measurements, and standardized anthropometry measures, briefly described in the following.

BioBeh score variables included WC, total serum cholesterol, fasting blood glucose, smoking, sleep duration, physical activity, perceived stress, and diet quality. Beh score variables included only smoking, physical activity, sleep, diet, and perceived stress.

#### 2.4.1 Data collection: lifestyle (behavioral) measures

##### 2.4.1.1 Physical activity, sleep, and stress

Physical activity was assessed using the Arabic Global Physical Activity Questionnaire (GPAQ-v2), with total activity calculated as minutes per week (24). Sleep (hours/24 h) was recorded using the validated Arabic version of the Pittsburgh instrument (25). Perceived stress was assessed using the validated Arabic version of the Cohen Perceived Stress Scale (PSS-10) (26), with higher scores indicating elevated stress.

##### 2.4.1.2 Dietary intake and quality

Usual dietary intake was assessed using a Saudi-specific food frequency questionnaire (FFQ) of 133 foods and beverages, including traditional Saudi dishes (27). Participants also completed two non-consecutive 24-h dietary recalls: one at recruitment and one by telephone using the multiple-pass method and standard food models (at recruitment) to estimate portion sizes (28). Trained dietitians administered the 24-h recalls. Dietary data from both the FFQ and recalls were analyzed using ESHA Food Processor SQL Software (ESHA Research, Salem, OR, USA). When traditional Saudi recipes were not available in the database, the team added them from a classic Saudi cookbook and, when needed, reached consensus on the most appropriate classification for items without a direct match in the food composition tables. Correlations between dietary macronutrients (e.g., protein and fat) from the FFQ and the mean of the two 24-h recalls were high (*r* = 0.52 and 0.61, respectively), indicating good agreement between the two dietary assessment methods in assessing participants' intakes.

Dietary data from the two 24-h recalls were used to assess diet quality as quantified by the Nutrient-Rich Food 9.3 (NRF9.3) index (29). The NRF9.3 score is a validated nutrient profiling score that evaluates overall nutritional quality of foods or diets by summing nine “nutrients to encourage” (i.e., protein, fiber, vitamins A, C, and E, calcium, iron, potassium, and magnesium) and subtracting three “nutrients to limit” (i.e., saturated fat, added sugars, and sodium) per 100 kcal. A higher NRF9.3 score indicates better nutritional quality per 100 kcal.

#### 2.4.2 Data collection: clinical (biological) measures

##### 2.4.2.1 Biological samples

Fasting blood samples were collected for fasting plasma glucose (FPG) and total cholesterol using standard enzymatic methods (30) at the study laboratories. About 10 mL of blood was drawn after an 8–10 h fast and immediately processed (centrifuged at 1,200 ×  g; plasma/serum aliquoted and stored at −80 °C). All biochemistry analyses were completed in a single lab visit, and results were provided to participants.

##### 2.4.2.2 Anthropometric measures

Anthropometry was measured by trained staff following a standard study protocol. Participants were asked to remove shoes and heavy clothing. Weight was recorded to the nearest 0.1 kg (InBody 570, South Korea) and height to the nearest 0.5 cm (Seca 274, Germany). The body mass index (BMI) was then calculated by dividing an individual's weight in kilograms by the square of their height in meters. WC was measured at the midpoint between the lowest rib and the iliac crest using a non-stretch fiberglass tape (nearest 0.5 cm) (31). All measures were collected twice, and if readings differed by >0.5 kg (weight) or >0.5 cm, a third reading was taken, and the mean of the two closest values was used.

##### 2.4.2.3 Blood pressure

BP was measured by trained staff in a quiet room. Participants were asked to avoid smoking, caffeine, and exercise for 30 min, to empty the bladder, and to sit quietly for 3–5 min with feet flat on the floor (2). Using the Omron M6 Comfort device, three consecutive readings were collected (2), and the mean of the readings was used in the analysis. Following the guidelines of the American College of Cardiology/American Heart Association (ACC/AHA), elevated BP was defined as a systolic BP (SBP) ≥ 120 mm Hg and a diastolic BP (DBP) < 80 mmHg, while hypertension was defined as SBP ≥130 mmHg or DBP ≥80 mmHg (2).

#### 2.4.3 Scoring framework

Building on previous work for score development (21), we classified each component as poor, intermediate, or ideal and assigned scores of 0, 1, or 2 points for risk, respectively, so that higher totals indicated lower risk. Here, “poor, intermediate, and ideal” refer to *a priori* health levels for each component defined from established clinical and public health thresholds: behaviors follow AHA Impact Goals; WC uses sex/ethnicity-specific cut-offs; physical activity uses AHA assessment guidance; sleep uses the American Academy of Sleep Medicine consensus recommendations; stress follows the PSS-10 questionnaire; and diet quality uses published NRF9.3 categories from a comparable UK sample. Exact thresholds for each component are provided in Table 1.

**Table 1:** Definition of poor, intermediate, and ideal BP lifestyle scores for each factor.

Goal/factor	Poor	Intermediate	Ideal
Biological factors			
Waist circumference (19, 32)	Male: >102 cm, Female: >88 cm (White); Male: >90 cm, Female: >88 cm (other ethnic groups)	Male: ≥94 to ≤102 cm, Female: ≥80 to ≤88 cm (White); Male: ≥85 to ≤90 cm, Female: ≥80 to ≤88 cm (other ethnic groups)	Male: <94 cm, Female: <80 cm (White); Male: <85 cm, Female: <80 cm (other ethnic groups)
Total serum cholesterol (19)	≥240 mg/dL	200–239 mg/dL	<200 mg/dL
Fasting blood glucose (19)	≥126 mg/dL	100–125 mg/dL	<100 mg/dL
Behavioral factors			
Smoking status (19)	Current	Former < 12 months	Never or quit ≥ 12 months
Sleep duration (33)	5 h or less or 9 h or more	6 h	7 and 8 h
Physical activity (34)	None	Recreational walking 1 to 8 h a week or practicing physical activity and sports activities for 1–4 h a week	Recreational walking for 8 h or more a week or practicing physical activity and sports activities for more than 4 h a week
Perceived stress scale (PSS-10) (26)	≥27	26–14	≤13
Diet quality (NRF 9.3) score (21)	≤16	16–25	>25

Each component is coded 0 (poor), 1 (intermediate), or 2 (ideal) so that higher values reflect healthier levels. Variables for which higher values indicate worse health [waist circumference (WC), total cholesterol, fasting plasma glucose, and perceived stress (PSS-10)] are reverse-coded relative to their raw direction; that is, a lower component score corresponds to a higher (worse) value (e.g., a lower WC score means a larger WC measurement). Diet quality (NRF9.3) and physical activity are coded in the same direction as the raw measures (higher is better). Sleep is scored by proximity to 7–8 h/night (7–8 = ideal; 6 = intermediate; ≤5 or ≥9 = poor).

### 2.5 Statistical analysis

Continuous variables are presented as mean (SD) and categorical variables as percentages. Participants were stratified into tertiles of the BioBeh score using the observed distribution (Q1: 0–9, Q2: 10–11, Q3: 12–16). These tertiles are empirical and were used for descriptive purposes, consistent with common epidemiologic practice when no validated clinical cut points exist for a continuous or composite exposure. Differences across tertiles were evaluated with generalized linear models (PROC GLM) for continuous variables and *χ*^2^ tests for categorical variables.

Two exposure constructs were examined: (i) the overall lifestyle risk score that includes biological and behavioral factors [BioBeh score, range (0–16)] and (ii) the behavioral-only health score [Beh score, range (0–10)]. For all scores and components, coding was consistent so that higher values reflected healthier levels.

Associations of lifestyle scores (BioBeh, Beh, and each component) with SBP and DBP were estimated using multivariable linear regression and expressed as mean differences (95% CI) per 1-point higher score. Associations of the scores with elevated BP and hypertension were estimated using logistic regression and expressed as odds ratios (ORs, 95% CI) per 1-point higher score. The lifestyle scores were entered as continuous variables on a per 1-point scale, assuming an approximately linear association over the observed range. We report two models. Model 1 was adjusted for age and sex. Model 2 was additionally adjusted for marital status, income, educational level, family history of hypertension, and antihypertensive medication use. These covariates were selected *a priori* based on plausible confounding reported in the literature. We treated the lifestyle score components themselves (WC, FPG, total cholesterol, smoking, physical activity, diet, sleep, and perceived stress) as exposures rather than adjustment variables to avoid overadjustment for intermediates on the causal pathway (35–39). Statistical significance was defined as a two-sided *p* < 0.05. Analyses were performed using SAS 9.3.

## 3 Results

### 3.1 Demographic and lifestyle characteristics

In total, *n* = 1,041 Saudi adults [31.8 (12.2) years] were included in the analyses, 38.1% of whom were males. The mean SBP/DBP was 111.5 (15.7)/75.0 (15.3) mmHg (Supplementary Table S1). The BioBeh score averaged 10.0 (2.0) points, whereas the Beh score averaged 5.0 (2.0) points.

Participants with higher BioBeh scores displayed a more favorable cardiometabolic profile, including lower BMI and WC, lower BP, and lower prevalence of hypertension compared to participants with lower scores (Table 1). In contrast, the prevalence of elevated BP was similar across score groups.

### 3.2 Association between lifestyle scores and BP

A 1-unit higher BioBeh score was significantly associated with lower SBP/DBP (Table 2). In fully adjusted models (Model 2), SBP was lower by 1.68 mmHg (95% CI: −2.58, −0.79) and DBP by 1.67 mmHg (95% CI: −2.66, −0.68).

**Table 2:** Baseline characteristics across tertiles of the BioBeh score (*n* = 1,041).

Parameters	Q1 (0–9)	Q2 (10–11)	Q3 (12–16)	*P*
*N*	347	347	347	
BioBeh score (Median)	7	10	12	
Beh score	3.5 (3.39, 3.61)	5.01 (4.85, 5.17)	6.54 (6.43, 6.66)	<0.0001
Men	42.2	33.3	36.0	0.06
Age (years)	34.22 (33.10, 35.34)	30.88 (29.22, 32.55)	29.45 (28.26, 30.65)	<0.0001
Marital status				
Married	46.4	58.2	68.0	0.08
Single	7.2	3.5	5.1	
Divorced	2.7	1.0	0.5	
Education				
High school	22.4	28.9	24.1	0.08
Bachelor's degree	50.5	49.3	54.6	
Postgraduate degree	13.9	13.4	14.7	
Income				
Less than 5,000	9.0	7.5	5.8	0.003
Between 5,000 and 10,000	27.4	20.9	19.0	
Between 10,000 and 20,000	36.1	37.3	34.0	
Above 20,000	26.0	33.3	39.9	
Smoking status				
Current	19.7	14.4	7.4	0.002
Family history of HTN				
Yes	76.2	72.1	76.4	0.47
SBP (mm Hg)	114.71 (113.43, 116)	111.3 (109.38, 113.21)	111.85 (110.47, 113.23)	0.002
DBP (mm Hg)	76.52 (75.13, 77.9)	76.17 (74.1, 78.23)	73.12 (71.64, 74.61)	<0.0001
Elevated BP	8.5	7.0	8.6	0.76
Hypertension	39.7	27.9	22.1	<0.0001
Antihypertensive medication use	9.1	8.3	8.6	0.92
BMI (kg/m^2^)	28.98 (28.47, 29.5)	25.92 (25.15, 26.69)	25.08 (24.52, 25.63)	<0.0001
Waist circumference (cm)	92.37 (91.16, 93.57)	84.53 (82.72, 86.33)	81.7 (80.41, 83)	<0.0001

Data presented as mean (SD) for continuous variables and % for frequency. BMI, body mass index; DBP, diastolic blood pressure; HTN, hypertension; PSS, perceived stress scale; SBP, systolic blood pressure. BioBeh score variables included WC, total serum cholesterol, fasting blood glucose, smoking, sleep duration, physical activity, perceived stress, and diet quality. BioBeh score, a combined score of behavioral and biological indices.

In contrast, the Beh score was not significantly associated with either SBP or DBP in adjusted models. Component-wise, WC score showed the largest inverse association (lower WC score = higher WC measure) with both SBP [−3.80 (95% CI: −4.70, −2.91)] and DBP [−3.31 (95% CI: −4.30, −2.32)] mm Hg; fasting glucose and total cholesterol (both reverse-coded) were related inversely to SBP at −1.82 (95% CI: −2.67, −0.97) and −1.07 (95% CI: −0.22, −1.91), respectively. Sleep, physical activity, perceived stress, smoking, and diet quality were not associated with BP after adjustment.

Neither the BioBeh score nor the Beh score showed a statistically significant association with elevated BP in either model (Table 3). Only borderline 19% lower odds of elevated BP were observed for higher fasting glucose score.

**Table 3:** Associations of lifestyle risk scores and components with systolic and diastolic blood pressure (*n* = 1,041).^a^

Variable	SBP	DBP
	Difference (95% CI), mmHg	Difference (95% CI), mmHg
BioBeh score		
Model 1	−1.43 (−2.30, −0.57)	−1.65 (−2.59, −0.72)
Model 2	−1.68 (−2.58, −0.79)	−1.67 (−2.66, −0.68)
Beh score		
Model 1	−0.12 (−0.98, 0.74)	−0.74 (−1.67, 0.19)
Model 2	−0.17 (−1.08, 0.74)	−0.75 (−1.75, 0.25)
Waist circumference score		
Model 1	−3.74 (−4.64, −2.84)	−3.54 (−4.52, −2.57)
Model 2	−3.80 (−4.70, −2.91)	−3.31 (−4.30, −2.32)
Smoking score		
Model 1	0.27 (−0.62, 1.17)	0.10 (−0.96, 0.96)
Model 2	0.30 (−0.58, 1.17)	0.08 (−0.88, 1.04)
Physical activity score		
Model 1	0.03 (−0.80, 0.87)	−0.28 (−1.18, 0.61)
Model 2	0.01 (−0.96, 0.98)	−0.60 (−1.66, 0.46)
Cholesterol score		
Model 1	−1.28 (−2.14, −0.42)	−0.53 (−1.45, 0.40)
Model 2	−1.07 (−1.91, −0.22)	−0.44 (−1.36, 0.36)
Fasting blood glucose score		
Model 1	−1.85 (−2.71, −0.99)	0.22 (−0.72, 1.16)
Model 2	−1.82 (−2.67, −0.97)	0.22 (−0.72, 1.16)
PSS score		
Model 1	−0.56 (−1.41, 0.30)	0.09 (−0.83, 1.01)
Model 2	−0.37 (−1.22, 0.48)	0.31 (−0.62, 1.24)
Sleep score		
Model 1	0.30 (−0.54, 1.14)	−0.35 (−1.26, 0.56)
Model 2	0.23 (−0.59, 1.05)	−0.35 (−1.25, 0.55)
NRF9.3 score		
Model 1	−0.39 (−1.25, 0.46)	−0.99 (−1.91, −0.08)
Model 2	−0.31 (−1.16, 0.54)	−0.77 (−1.70, 0.16)

a Values are mean (95% CI) differences. Negative values indicate lower BP with higher lifestyle scores. The following components are reverse-coded: waist circumference, cholesterol, fasting blood glucose, and PSS. Model 1 was adjusted for age and sex. Model 2 was adjusted for Model 1 + marital status, income, level of education, family history of hypertension, and antihypertensive medication use. DBP, diastolic blood pressure; NRF9.3, nutrient rich food index; PSS, perceived stress scale; SBP, systolic blood pressure. BioBeh score variables included WC, total serum cholesterol, fasting blood glucose, smoking, sleep duration, physical activity, perceived stress, and diet quality. Beh score included only smoking, physical activity, sleep, diet, and perceived stress. Beh score, a behavioral-only lifestyle risk score; BioBeh score, a combined score of behavioral and biological indices.

In fully adjusted models (Model 2), each one-point increase in the BioBeh score was associated with a 27% lower odds of hypertension (OR: 0.73; 95% CI: 0.63, 0.85). In contrast, the behavioral-only lifestyle risk score was not significantly associated with hypertension (OR: 0.89; 95% CI: 0.76, 1.03) (Table 4). Among individual components, the waist circumference score was associated with 43% lower odds of hypertension [OR 0.57 (95% CI: 0.49, 0.66)].

**Table 4:** Odds of elevated BP per 1-point increase in lifestyle scores and components (*n* = 1,041).^a^

Variable	Elevated BP	
	Model 1	Model 2
Index	OR (95% CI)	OR (95% CI)
BioBeh score	0.97 (0.77, 1.22)	0.89 (0.69, 1.14)
Beh score	1.06 (0.85, 1.33)	1.00 (0.78, 1.28)
Waist circumference score	0.95 (0.74, 1.22)	0.88 (0.68, 1.14)
Smoking score	1.14 (0.92, 1.42)	1.13 (0.91, 1.40)
Physical activity score	1.07 (0.85, 1.35)	1.13 (0.85, 1.52)
Cholesterol score	0.97 (0.76, 1.25)	0.99 (0.77, 1.27)
Fasting blood glucose score	0.80 (0.65, 0.98)	0.81 (0.66, 1.00)
PSS score	0.88 (0.70, 1.11)	0.83 (0.66, 1.05)
Sleep score	1.05 (0.83, 1.31)	1.05 (0.83, 1.32)
NRF9.3 score	1.04 (0.83, 1.31)	1.05 (0.83, 1.32)

a Values are odds (95% CI) differences per 1-point increase in the score. Negative values indicate lower BP with higher lifestyle scores. The following components are reverse-coded: waist circumference, cholesterol, fasting blood glucose, and PSS. Model 1 was adjusted for age and sex. Model 2 was adjusted for Model 1 + marital status, income, level of education, family history of hypertension, and antihypertensive medication use. BP, blood pressure; NRF9.3, nutrient rich food index; PSS, perceived stress scale. BioBeh score variables included WC, total serum cholesterol, fasting blood glucose, smoking, sleep duration, physical activity, perceived stress, and diet quality. Beh score included only smoking, physical activity, sleep, diet, and perceived stress. Beh score, a behavioral-only lifestyle risk score; BioBeh score, a combined score of behavioral and biological indices.

**Table 5:** Odds of hypertension per 1-point increase in lifestyle scores and components (*n* = 1,041)^a^.

Variable	Hypertension	
	Model 1	Model 2
Index	OR (95% CI)	OR (95% CI)
BioBeh score	0.76 (0.66, 0.88)	0.73 (0.63, 0.85)
Beh score	0.91 (0.79, 1.05)	0.89 (0.76, 1.03)
Waist circumference score	0.56 (0.48, 0.65)	0.57 (0.49, 0.66)
Smoking score	1.06 (0.92, 1.23)	1.07 (0.92, 1.24)
Physical activity score	0.96 (0.84, 1.11)	0.90 (0.76, 1.06)
Cholesterol score	1.02 (0.89, 1.17)	1.00 (0.87, 1.15)
Fasting blood glucose score	1.05 (0.91, 1.21)	1.05 (0.91, 1.21)
PSS score	0.91 (0.79, 1.05)	0.94 (0.81, 1.09)
Sleep score	0.96 (0.83, 1.11)	0.96 (0.83, 1.11)
NRF9.3 score	0.91 (0.79, 1.06)	0.94 (0.81, 1.09)

a Values are odds (95% CI) differences per 1 point increase in the score. Negative values indicate lower BP with higher lifestyle scores. The following components are reverse-coded: waist circumference, cholesterol, fasting blood glucose, and PSS. BioBeh score variables included WC, total serum cholesterol, fasting blood glucose, smoking, sleep duration, physical activity, perceived stress, and diet quality. Beh score included only smoking, physical activity, sleep, diet, and perceived stress. Model 1 was adjusted for age and sex. Model 2 was adjusted for Model 1 + marital status, income, level of education, family history of hypertension, and antihypertensive medication use. BP, blood pressure; NRF9.3, nutrient rich food index; PSS, perceived stress scale. Beh score, a behavioral-only lifestyle risk score; BioBeh score, a combined score of behavioral and biological indices.

## 4 Discussion

### 4.1 Principal findings

In this sample of Saudi adults, a higher overall lifestyle score that includes behavioral and biological factors was associated with lower SBP and DBP and lower odds of hypertension. In contrast, a behavior-only score showed no significant relationship with any BP outcomes. Among individual components, WC, a measure of central adiposity (10), showed the strongest association with BP outcomes. This pattern is expected in cross-sectional analyses because biological intermediates (central adiposity, lipids, and fasting glucose) are more proximal to BP pathophysiology and are measured more objectively than self-reported behaviors, which carry greater measurement error. Our study demonstrates this gradient in a Middle Eastern cohort and shows that a biologically enriched composite captures BP and hypertension more strongly than a behavior-only score, extending prior UK Airwave findings (21) to a new population and highlighting central adiposity as a principal driver.

### 4.2 Comparison with prior literature

Our findings align with and add geographic diversity to evidence that biologically enriched composite lifestyle phenotypes relate meaningfully to BP. In our data, a behavior-only score was not associated with SBP/DBP or hypertension, while a score that includes biological intermediates was inversely related to both outcomes. In the UK Airwave cohort (>40,000 adults), each 1-point increase in a “basic” lifestyle score (WC, smoking, total cholesterol) was associated with ∼2 mmHg lower SBP and DBP and ∼28% lower odds of hypertension. Adding sleep, physical activity, and diet modestly attenuated but did not abolish the associations (21). Our study demonstrates a similar pattern with stronger associations for a score that includes biological intermediates, and specifically highlights central adiposity as a major driver, consistent with the Airwave study. Differences in the magnitude of effects between the current and previous study are expected, given population composition (age 31.8 vs. 40.5 years, respectively), lifestyle score definitions, and hypertension threshold (we used ACC/AHA ≥130/80 mmHg; the UK Airwave study used ≥140/90 mmHg or diagnosis/antihypertensive medication) (21). The Airwave “basic” score comprised WC, smoking, and total cholesterol with sleep, physical activity, diet, and alcohol added sequentially. Our BioBeh score included WC, total cholesterol, fasting glucose, smoking, sleep, physical activity, perceived stress, and diet. We also examined a behavior-only score (smoking, sleep, physical activity, perceived stress, and diet).

Our results are also consistent with broader frameworks, such as the AHA's Life's Essential 8, which emphasizes joint contributions of behaviors (diet, physical activity, tobacco exposure, and sleep) and biological factors (adiposity, lipids, glucose, and BP) to cardiovascular health and incident hypertension (20). Randomized controlled trials and meta-analyses further support that healthy dietary patterns and sodium reduction lower BP (12, 13). However, in cross-sectional analyses, self-reported behaviors like the ones reported in our study are measured with more error and reflect only a moment in time. Biological markers, such as WC, lipid levels, and fasting glucose, show the accumulated effects of lifestyle over time and are measured more objectively. As a result, composite scores that include these biological markers tend to show stronger and clearer associations with BP than scores based only on reported behaviors (20).

We observed no associations for elevated BP but robust associations for hypertension. Several factors may explain this finding. The ACC/AHA criteria for elevated BP (SBP 120–129 mmHg/DBP < 80 mmHg) define a narrower phenotype that is less prevalent in younger samples, limiting power to detect associations. Furthermore, behavioral and biological exposures may exert stronger effects on diastolic BP captured within the hypertension definition (≥130/80 mmHg) than on isolated systolic elevation with normal DBP (40).

### 4.3 Potential mechanisms

Excess visceral and central adiposity elevates BP through various pathways, including activation of the sympathetic nervous system and renin–angiotensin–aldosterone system, renal sodium retention, endothelial dysfunction, and low-grade inflammation (41, 42). Dyslipidemia may relate to higher SBP partly through arterial stiffness, with total cholesterol acting as a proxy for adverse lipid profile and vascular remodeling (9, 43). Improved fasting glucose levels are also linked to lower BP through reduced insulin resistance, improved endothelial function, and attenuation of sodium retention (43, 44). The superiority of the WC component in our findings is therefore biologically consistent and reflects large cohort evidence that central adiposity is strongly linked to BP and incident hypertension even among individuals with normal BMI.

### 4.4 Public health and clinical implications

Our results support risk screening strategies that emphasize central adiposity. In primary care and community settings, WC, an inexpensive, portable measure, can be used for first-level assessment. Where recent basic labs (fasting glucose and a lipid profile) are already available from routine care, these data can help prioritize individuals for intensive lifestyle counseling and early BP management (45, 46), complementing current guideline recommendations that encourage comprehensive lifestyle modification at the core of care (8, 20).

The pattern we observed (behavior-only score null; biologically enriched score associated with BP) is consistent with behaviors exerting their effects through biological intermediates. Although diet quality and physical activity were not independently related to BP in our models, we include them here as modifiable factors with trial evidence for lowering BP and central adiposity. For management, it is more precise to target central adiposity (WC) rather than “weight” alone. Effective strategies include sustained energy deficit achieved through a dietary pattern rich in fiber and minimally processed foods, with restricted intake of added sugars, refined carbohydrates; regular physical activity; and counseling on adequate sleep and stress management. Collectively, these strategies preferentially reduce visceral adiposity and WC and are feasible within routine primary care counseling.

### 4.5 Strengths and limitations

The cross-sectional design prevents causal inference and is subject to reverse causation. It is possible that individuals with higher BP may have altered their behaviors, which could attenuate observed associations for the behavioral-only score. Conversely, elevated or undiagnosed hypertension, or related comorbid conditions, may worsen perceived stress, sleep, and some health behaviors. Furthermore, self-reported behaviors are prone to recall error and misclassification. In addition, participants were recruited using convenience sampling through social media, university email lists, primary care clinics, and mall-based portable clinics in Riyadh and Almadinah. This approach likely selected younger, more educated, and higher-income adults with greater health awareness. Therefore, findings may not be generalizable to the broader Saudi population, particularly those living in rural areas or with lower socioeconomic status. Despite extensive multivariable adjustment for sociodemographic and other factors (e.g., age, sex, marital status, education, and income), residual confounding cannot be excluded, including unmeasured or imprecisely measured factors such as lipid-lowering therapy, sodium intake, and occupational stress (47). Because BP was recorded at a single visit, we could not capture visit-to-visit variability or white-coat effects, so some regression dilution and imprecision may remain. Finally, we did not develop or validate a prediction model, so measures such as ROC curves or AUC are beyond the scope of this analysis; future work should formally assess the discrimination and calibration of lifestyle-based scores for hypertension risk.

Strengths of this study include repeated BP measurement (three readings averaged with an automated device), use of validated tools in Arabic for physical activity, sleep, and perceived stress, and a detailed dietary assessment that combined recalls and a nutrient density index. The lifestyle scores were specified *a priori* from established frameworks and our prior work, but with adaptations for this setting: The behavior-only score included smoking, physical activity, sleep, diet, and perceived stress; the overall (bio + behavior) score additionally included WC, total cholesterol, and fasting glucose.

Prospective validation is needed to test whether the composite score predicts incident hypertension and BP trajectories in Saudi adults and to examine whether component weighting (e.g., giving central adiposity greater weight) improves discrimination. Implementation studies should assess whether including this phenotype in screening programs improves detection and BP control, as highlighted by the WHO's 2023 report (7).

## 5 Conclusion

A lifestyle risk score that integrates biological intermediates, particularly WC, captures meaningful variation in BP and hypertension in Saudi adults, whereas behavior-only scores show weaker associations. These findings underscore the role of central adiposity and related metabolic factors as key targets for prevention and risk stratification in this population. Policies and clinical programs that directly reduce central adiposity and improve lipid and glucose profiles may yield the greatest population-level reductions in BP.

## Data Availability

The raw data supporting the conclusions of this article will be made available by the authors, without undue reservation.
