# Supplementary material for: Beyond behaviors: do biological intermediates improve lifestyle scoring for blood pressure?
Source: Front Cardiovasc Med. 2026 Jan 13;12:1713086. doi: 10.3389/fcvm.2025.1713086 (PMC12835251; doi:10.3389/fcvm.2025.1713086)
Supplement: Supplementary file 1 [file Datasheet1.docx]

Supplementary Material

Assessed for eligibility (n=1,204)

## Enrollment

Excluded (n=163):

- Did not complete questionnaires and/or body measurements (n=28)
- Pregnant or lactating women (n=4)
- Used nutritional supplements of minerals and vitamins, antihypertensive medications, corticosteroid medications, antidepressant medications, hormone therapy (n=14)
- Had a previous history of cardiovascular disease, stroke, T1DM, or dyslipidemia (n=56)
- Energy consumption below 500 kcal/d and above 6,000 kcal/d (n=26)
- Withdrew (n=35)

Included (n= 1,041)

**Supplementary Figure 1. Flow Diagram**

| **Supplementary Table 1. Baseline characteristics of study participants (n=1,041)** | |
| --- | --- |
| **Variables** |  |
| n | 1041 |
| Men | 38.1 |
| Age (years) | 31.8 (12.2) |
| Moderate physical activity (min/week) | 254.2 (433.1) |
| Marital status |  |
| Married | 35.93 |
| Single | 56.87 |
| Divorced | 7.2 |
| Education |  |
| High school | 28.1 |
| Bachelor's degree | 57.8 |
| Postgraduate degree | 14.1 |
| Income |  |
| Less than 5,000 | 8.5 |
| Between 5,000 to 10,000 | 23.0 |
| Between 10,000 to 20,000 | 35.5 |
| Above 20,000 | 33.0 |
| Smoking status |  |
| Current | 14.0 |
| Family history of hypertension |  |
| Yes | 75.5 |
| SBP (mm Hg) | 111.53 (15.7) |
| DBP (mm Hg) | 75.00 (15.3) |
| Elevated BP | 8.3 |
| Hypertension | 30.7 |
| BMI (kg/m^2^) | 26.8 (6.2) |
| Waist circumference (cm) | 84.9 (17.3) |
| lifestyle score-BioBeh | 10.0 (2.0) |
| lifestyle score-Beh | 5.0 (2.0) |
| Waist circumference score | 1.3 (0.9) |
| Smoking score | 0.9 (0.3) |
| Physical activity score | 1.1 (0.9) |
| Cholesterol score | 1.7 (0.6) |
| Fasting blood glucose score | 1.8 (0.5) |
| PSS score | 1.2 (0.6) |
| Sleep score | 0.7 (0.8) |
| NRF9.3 score | 1.0 (0.8) |
| Note: Data presented as Mean (SD) for continuous variables and % for frequency. Abbreviations: Body mass index (BMI); Diastolic blood pressure (DBP); Nutrient Rich Food Index (NRF9.3); Perceived stress scale (PSS); Systolic blood pressure (SBP). **Lifestyle score-BioBeh variables included: WC, total serum cholesterol, fasting blood glucose,** smoking, sleep duration, physical activity, perceived stress, and diet quality. The Lifestyle score-Beh included **only** smoking, physical activity, sleep, diet, and perceived stress. | |
